# Characterisation of *Dichelobacter nodosus* on Misshapen and Damaged Ovine Feet: A Longitudinal Study of Four UK Sheep Flocks

**DOI:** 10.3390/ani11051312

**Published:** 2021-05-03

**Authors:** Caroline M. Best, Janet Roden, Kate Phillips, Alison Z. Pyatt, Tristan Cogan, Rosemary Grogono-Thomas, Malgorzata C. Behnke

**Affiliations:** 1 Department of Veterinary Health & Animal Sciences, Harper Adams University, Newport, Shropshire TF10 8NB, UK; jroden@harper-adams.ac.uk (J.R.); kphillips@harper-adams.ac.uk (K.P.); mbehnke@harper-adams.ac.uk (M.C.B.); 2 International Office, Veterinary Medicines Directorate, Addlestone, Surrey KT15 3NB, UK; a.pyatt@vmd.gov.uk; 3 School of Veterinary Sciences, University of Bristol, Langford, Bristol BS40 5DU, UK; tristan.cogan@bristol.ac.uk (T.C.); R.Grogono-Thomas@bristol.ac.uk (R.G.-T.)

**Keywords:** *Dichelobacter nodosus*, footrot, sheep, subclinical carriers, hoof conformation, infection, disease challenge

## Abstract

**Simple Summary:**

The bacterium *Dichelobacter nodosus* is the cause of footrot in sheep: a painful, contagious foot disease. Some sheep may be more susceptible to carrying *D. nodosus* on their feet, despite showing no signs of disease, and pose a risk to flock health and welfare. This study investigated whether misshapen or damaged feet (poor hoof conformation) were more likely to have *D. nodosus* present and in greater quantities than feet in good condition. Eighty-five ewes from four flocks were examined three times, on average, across a 12-month period. Poor hoof conformation traits were observed in over 92% of foot observations. Feet with poor sole and heel conformation were more likely to have higher quantities of *D. nodosus* than those in good conformation. Furthermore, on feet positive for *D. nodosus*, wall overgrowth contributed towards higher *D. nodosus* load. We highlight feet with aspects of poor conformation traits to covertly harbour *D. nodosus*. These feet could transmit *D. nodosus* to other feet of sheep. Increasing our understanding of *D. nodosus* infection is crucial in helping farmers improve management practices to reduce footrot.

**Abstract:**

*Dichelobacter nodosus* is the causal agent of ovine footrot, a contagious disease of welfare and economic concern worldwide. Damaged feet may be subclinical carriers of *D. nodosus* and covertly spread infection. Accordingly, we evaluated the risk of misshapen and damaged feet on *D. nodosus* presence and load in four commercial UK sheep flocks. Foot-level observations and swabs (*n* = 972) were collected from ewes (*n* = 85) over 12 months. On average, ewes were sampled three times. Feet were inspected for disease and scored (good/poor) for three hoof conformation traits (sole and heel, wall, and wall overgrowth). Swabs were analysed for presence and load of *D. nodosus*, and mixed models were constructed. Poor hoof conformation traits were present in 92.5% of foot-level observations. Feet with poor sole and heel conformation were more likely to have higher *D. nodosus* loads (β = 0.19, 95% CI: 0.04–0.35) than those with good conformation. Furthermore, on feet positive for *D. nodosus*, wall overgrowth was associated with higher *D. nodosus* loads (β = 0.27, 95% CI: 0.01–0.52). Feet with aspects of poor conformation covertly harbour *D. nodosus* and are a source of infection. Flock management should be guided by hoof conformation to reduce disease challenge.

## 1. Introduction

Ovine footrot is a contagious disease of significant concern to sheep producers worldwide [1]. In England, footrot is the principal cause of lameness in sheep and affects >70% of flocks [2]. Footrot has two distinct clinical presentations reflecting a continuum of disease severity: interdigital dermatitis (ID), characterised by the inflammation of the interdigital skin, and severe footrot (SFR), characterised by separation of the keratinous hoof horn from underlying tissue [3]. Accordingly, footrot has severe implications on the health [4], welfare [5], and productivity [6,7,8], of sheep. Associated treatment and production losses are estimated to cost the UK sheep industry £24 to £80 million per year [8,9].

The causal agent of footrot (both clinical presentations), *Dichelobacter nodosus*, is a gram-negative, obligate anaerobe of the ovine foot. Whilst *D. nodosus* is found on healthy and diseased feet, load of *D. nodosus* is highest and most persistent on footrot-affected feet, suggestive of its key role in the initiation of ID and progression of SFR [10,11]. Therefore, diseased feet are the primary source of infection, and indirect transmission can occur readily via contaminated ground where *D. nodosus*-positive feet are present [11,12]. Damage to the interdigital skin is essential for invasion of *D. nodosus* into the hoof epidermis and for disease to initiate [3]. Environmental factors such as rainfall [13], pasture quality [14], and soil type [12], have been implicated in mediating clinical development of footrot by increasing the probability of a foot becoming damaged [15]. Secondary pathogens, such as *Fusobacterium necrophorum*, opportunistically colonise footrot-affected feet and increase disease severity [11].

It is understood that not all sheep are equally susceptible to footrot at all times [16]. Host factors have been implicated in the inherent susceptibility of sheep developing clinical footrot [1,14]. The primary role of hoof horn is to protect sensitive internal structures from the external environment [13]. Feet with poor conformation, such as damaged or misshapen hoof horn, may be at risk of invasion by *D. nodosus* [3]. This provides some explanation as to why sheep with misshapen or damaged feet are more likely to develop clinical footrot and become lame [14]. However, no studies have purposefully examined the dynamics of *D. nodosus* on misshapen or damaged feet, and given its importance in further elucidating disease pathogenesis, this warrants further investigation.

Feet with poor conformation are also thought to sequester infection [14]. Subclinical carriers of *D. nodosus*, such as clinically healthy feet with detectable *D. nodosus*, are a major concern to strategies controlling footrot in flocks [17]. These feet could shed substantial amounts of *D. nodosus* into the environment but remain unnoticed in the absence of clinical signs. Furthermore, conditions conductive for disease development, such as wet ground [18], could favour the recurrence of clinical disease in covert carriers of *D. nodosus*. Therefore, sustained eradication of *D. nodosus* at flock level is dependent upon identifying subclinical carriers. Although removal of footrot-affected sheep during periods of zero transmission (i.e., prolonged dry periods) could result in *D. nodosus* elimination [11], relapse of clinical disease could be mitigated by routine screening. Subclinical carriers of *D. nodosus* are identifiable by polymerase chain reaction (PCR) [19], but routine use of molecular diagnostics is costly, labour intensive, and time consuming, and unlikely to be implemented as routine practice on-farm. Identifying physical characteristics of apparently healthy feet that carry *D. nodosus* would be valuable to help recognise sheep at risk of shedding higher quantities of *D. nodosus* into the environment and acting as silent spreaders of infection within the flock.

To the best of the authors’ knowledge, no epidemiological studies have investigated the associations between hoof conformation and the presence and load of *D. nodosus*, nor identified the specific hoof conformation traits associated with the greatest risk of infection. This is integral to developing our understanding of host heterogeneity, namely hoof morphology, as a mechanism influencing the dynamics of *D. nodosus* infection on feet. The aim of this study was to evaluate the risk of misshapen and damaged feet on *D. nodosus* infection in commercial UK sheep flocks.

## 2. Materials and Methods

This study was reviewed and approved by the Harper Adams University Ethics Committee (0364-201808-PGMPHD).

### 2.1. Study Design and Farm Selection

The study was a longitudinal, repeated cross-sectional field survey of four commercial sheep farms (identified as A-D) in England and Wales. Farms were convenience-selected based on the following criteria: convenient location, known history of footrot, and willingness of farmers to participate. Farmers who routinely trimmed the feet of sheep and/or routinely footbathed were excluded from the selection, due to the putative association with hoof damage [20,21]. Farm and flock characteristics are summarised in Table 1. Farms were visited four times during the course of the study; September 2019 (Visit 1), January 2020 (Visit 2), July 2020 (Visit 3) and September 2020 (Visit 4).

### 2.2. Ewe Selection

Fifteen ewes were initially convenience sampled from each farm and marked for inclusion in the study. Ewes were individually identified by ear tag numbers. Due to the dynamic nature of commercial flocks, it was not always possible to sample the same ewe at repeated time points, as farmers continued to cull unproductive ewes regardless of inclusion in the study. Therefore, in the absence of ewes at a specific visit, previously unsampled ewes were included to achieve a minimum of 15 ewes per farm per visit, a similar strategy to [22]. All ewes were managed as part of the main flock throughout the study period.

### 2.3. Collection of Foot, Sheep and Flock Data

Ewes were visually inspected for lameness using a four-point locomotion scoring system [23] and scored for body condition using a 1–5 scale with 0.5 increments [24]. All four hooves of each ewe were first examined for the presence of ID and SFR, based on the description by [25]. Severity of disease was not recorded. All eight individual claws were then assessed for three hoof conformation parameters using four-point ordinal scoring systems, adapted from [14,26]: (1) sole and heel conformation, (2) hoof wall conformation, and 3) hoof wall overgrowth (Table 2). One digital photograph of each hoof was taken as a record. All data were recorded by a single observer (CMB) on paper recording sheets. Flock level data were obtained from management practice questionnaires completed by each farmer.

### 2.4. Collection of Swab Samples

One sterile cotton swab (Fisherbrand™ Swabs, Fisher Scientific, UK) was taken from the interdigital skin of each foot of each sheep sampled. The swab was swiped five times across the surface of the skin. Clean gloves were worn whilst sampling and changed between sheep. Swabs were stored dry in labelled 2 mL microcentrifuge tubes. Due to laboratory access restrictions, storage and subsequent processing of swabs varied between visits; swabs collected at visits 1 and 2 were stored at 4 °C for a maximum of 24 h before processing, whilst those collected at visits 3 and 4 were stored at −20 °C until processed.

### 2.5. DNA Extraction

Genomic DNA was extracted from swabs using the spin column method (DNeasy Blood and Tissue Kit, Qiagen Ltd., Manchester, UK). DNA extraction followed the manufacturer’s recommended protocol, with minor amendments. In short, 500 µL of nuclease-free water (Ambion^®^, Fisher Scientific, Loughborough, UK) was added to each swab tube and vortexed. Swabs were allowed to defrost or warm to room temperature, as appropriate, prior to addition of water. A 200 µL aliquot of sample suspension was mixed with 20 µL Proteinase K and 200 µL of Buffer AL, as provided. Following 10 min at 56 °C, 200 µL of ethanol was added to each sample, vortexed briefly and applied to a spin column, which was centrifuged at 10,000 rpm for 1 min. Wash and centrifugation steps with Buffer AW1 and AW2 followed, where the collection tube was discarded and replaced after each wash. Finally, 100 µL of elution Buffer AE was applied to each column. The resultant DNA was collected in a 2 mL microcentrifuge tube and stored at −20 °C. DNA quantity and quality were assessed using the Genova Nano spectrophotometer (Jenway, Stone, UK).

### 2.6. Detection of Dichelobacter nodosus on Swabs

To screen DNA samples for *D. nodosus*, a TaqMan^®^-based real-time PCR targeting the *D. nodosus*-specific fragment of the 16S rRNA gene was employed—an assay previously developed by [27]. The 16S rRNA gene exists in three copies in the *D. nodosus* genome [28], making it a robust assay for when sensitive detection is required [27]. Details of assay sensitivity and specificity have been described previously [27].

Each reaction (25 µL) contained Brilliant III ultra-fast qPCR master mix (Agilent Technologies, TX, USA) (12.5 µL), forward primer (1.0 µL), reverse primer (1.0 µL), TaqMan probe (1.0 µL), nuclease-free water (9.0 µL) and template DNA (1.0 µL). Details of primers and probe can be found in Supplementary Table S1. All PCR reactions were run in triplicate. Each real-time PCR plate also contained an internal positive control (*D. nodosus* strain VCS1703A) and a non-template control (nuclease-free water in place of template DNA), both run in triplicate.

Real-time PCR amplification was performed using the Stratagene Mx3005P real-time PCR system (Agilent Technologies, Santa Clara, CA, USA). Cycling conditions consisted of an initiation denaturation step at 95 °C for 10 min, followed by 40 cycles at 95 °C for 30 s and 60 °C for 1 min. Only DNA samples producing probe-specific fluorescent signals in all three of the triplicate reactions were considered positive for *D. nodosus*.

### 2.7. Quantification of Dichelobacter nodosus on Swabs

To quantify the load of *D. nodosus* present on swabs, a TaqMan-based qPCR assay targeting the 61 bp sequence within the *rpoD* gene (RNA polymerase sigma-70 factor alpha) of *D. nodosus* was employed—an assay previously developed by [29]. The *rpoD* gene exists as a single copy number in the *D. nodosus* genome and is therefore more suited to quantification than the 16S rRNA assay. Details of assay sensitivity and specificity have been described previously [10,29].

Only DNA samples positive for *D. nodosus*, as determined by the 16S rRNA assay, were tested. Each reaction (25 µL) contained Brilliant III ultra-fast qPCR master mix (Agilent Technologies, TX, USA) (12.5 µL), forward primer (2.25 µL), reverse primer (2.25 µL), TaqMan probe (0.625 µL), bovine serum albumin 10 mg mL^−1^ (2.5 µL), nuclease-free water (3.875 µL) and template DNA (1.0 µL). Details of primers and probe can be found in Supplementary Table S1. All PCR reactions were run in triplicate. Each real-time PCR plate included a set of 10-fold serial dilutions of plasmid DNA from the purified and cloned PCR product of the 61 bp *rpoD* insert of *D. nodosus* strain VCS1703A, ranging from approximately 10^7^ to 10^0^ *rpoD* copies µL^−1^. Plasmid standards were run in triplicate, in addition to non-template controls.

qPCR amplification was performed as described above, except cycling conditions consisted of 1 cycle at 50 °C for 2 min, 1 cycle at 95 °C for 10 min, followed by 40 cycles at 95 °C for 15 s and 55 °C for 1 min.

Load of *D. nodosus* (log_10_ + 1 *rpoD* copy number µL^−1^) from DNA samples was estimated from a standard curve obtained from the 10-fold serial dilution of plasmid DNA. Mean log_10_ + 1 *rpoD* copy number µL^−1^ was calculated from the reaction triplicate and used in subsequent analyses. Log_10_ + 1 resulted in all undetectable loads being coded as 0 on the log scale.

### 2.8. Data Preparation

All data were manually entered into Excel 2016 (Microsoft Corporation, Redmond, WA, USA).

For each hoof conformation variable, the total score was calculated at foot level by taking the sum of scores from the paired (lateral and medial) claws. For all three variables, a foot was defined as ‘good’ when the maximum score was 0 and ‘poor’ when the score was ≥1.

Clinical status had two descriptor codes, healthy and ID, as no additional clinical diseases were observed in the sample. The locomotion score was excluded from analyses as scores > 0 were rare. Details of all sheep- and foot-level variables are found in Table 3.

### 2.9. Statistical Analysis

All statistical analyses were performed in Genstat (VSN International, Hertfordshire, UK) and R statistical software version 3.5.3. Associations between categorical variables were investigated using Pearson Chi-Squared tests. Kruskal-Wallis tests were used to investigate associations between continuous and categorical variables. Probability values of <0.05 were considered significant.

#### 2.9.1. Associations with Clinical Status of Feet

To identify the association between hoof conformation and risk of a foot being affected by disease, univariable and multivariable binomial mixed effects models were constructed with the binary outcome variable ‘clinical status’, categorised as healthy or ID. Models were constructed using the “glmer” function from the “lme4” package in R [30]. To account for repeated observations over time of feet and feet clustered within sheep, ‘Ewe number’ was included as a random effect, alongside ‘Farm and Visit’ (e.g., A1; denotes farm A, visit 1) to account for ewes clustering within farms and the unobserved influence of factors (e.g., weather) changing over time, as well as the interaction between them. Fixed effects (i.e., sheep- and foot-level variables; Table 3) were first tested in univariable models. Variables with *p* < 0.2 were then used to develop the multivariable model, using a backward elimination procedure, until only significant variables remained. The optimal multivariable model was selected by examining Akaike Information Criteria (AIC) (the model with the lowest AIC was favoured) and R squared values, using the “performance” package in R. To control for collinearity, Variance Inflation Factors (VIF) were checked using the “car” package in R [31]; only variables with VIF < 5 were included in model building. Strength of association between clinical status and fixed effects were evaluated by calculating odds ratios from the coefficient returned by the model.

#### 2.9.2. Associations with Presence of *Dichelobacter nodosus* on Feet

To identify the association between hoof conformation and the risk of a foot being positive for *D. nodosus*, univariable and multivariable binomial mixed effects models were constructed with the binary outcome variable ‘presence of *D. nodosus*’, categorised as undetected or detected. Models were constructed using the “glmer” function as described above.

#### 2.9.3. Associations with Load of *Dichelobacter nodosus* on All Feet

To identify the association between hoof conformation and load of *D. nodosus* on all feet, univariable and multivariable linear mixed effects models were constructed with the continuous outcome variable ‘Load of *D. nodosus* on all feet’ (log_10_ + 1 *rpoD* genome copies μL^−1^). Models were constructed using the “lmer” function of the “lme4” package in R. Random factors were ‘Ewe number’ and ‘Farm and Visit’. The relative fit of models was assessed using AIC values, as before. Residual plots were inspected to identify outliers and to ensure the normality assumption was met.

#### 2.9.4. Associations with Load of *Dichelobacter nodosus* on Positive Feet

To identify the association between hoof conformation and the load of *D. nodosus* on positive feet only, univariable and multivariable linear mixed effects models were constructed with the continuous outcome variable ‘Load of *D. nodosus* on positive feet only’ (log_10_ + 1 *rpoD* genome copies μL^−1^, omitting feet with undetected loads coded as 0). Models were constructed using the “lmer” function as described above.

## 3. Results

### 3.1. Descriptive Results

#### 3.1.1. Study Sample

In total, 243 sheep-level and 972 foot-level observations, in addition to 972 swab samples, were obtained from 340 feet of 85 individual ewes on four farms across four visits. On average, individual ewes were sampled 2.9 times (95% CI: 2.6–3.2), although the majority were sampled four times (*n* = 40, 47.1%) (Table 4). The majority of ewes (61.2%, *n* = 52/85) were aged <4 years, and the remaining 38.8% (*n* = 33/85) were aged ≥4 years at the start of the study. On average, ewes were aged 3.3 years (95% CI: 3.1–3.6 [range: 1–8]).

#### 3.1.2. Hoof Conformation

The distribution of good and poor states of three hoof conformation parameters are presented in Table 5. The majority of foot-level observations had good sole and heel conformation (78.9%, *n* = 767/972) but poor wall conformation (75.2%, *n* = 731/972) and wall overgrowth (72.4%, *n* = 704/972).

Approximately 92% (*n* = 899/972) of foot-level observations scored poor for at least one hoof conformation variable, whereas 12.6% (*n* = 122/972) scored poor for all three conformation variables. A foot with poor wall conformation was more likely to have poor, rather than good, sole and heel conformation (*p* < 0.001) and wall overgrowth (*p* = 0.006). More poor wall overgrowth scores were recorded on front than back feet (*p* < 0.001). No difference in sole and heel conformation nor wall conformation scores were observed between front and back feet (*p* > 0.05).

#### 3.1.3. Clinical Status and Lameness

Of the 972 foot-level observations, 12.8% (*n* = 124/972) were affected by ID, and 87.2% (*n* = 848/972) remained healthy. ID was observed on all farms and across all visits. No other clinical diseases were recorded. Throughout the duration of the study, 67.9% (*n* = 231/340) of feet remained healthy. Of individual feet affected by ID (*n* = 109/340), 86.2% (*n* = 94/109) were affected for one visit only, whilst 13.8% (*n* = 15/109) from eight sheep were affected for two non-consecutive visits. No feet were affected with ID over consecutive visits, nor affected with ID for > two visits. No difference in clinical status between front and back feet was observed (*p* > 0.05).

Over the course of the study, lameness (locomotion score > 0) was observed in 5.9% (*n* = 5/85) of individual ewes. One ewe was lame twice at visit 1 and visit 3.

#### 3.1.4. Presence of *Dichelobacter nodosus*

Overall, *D. nodosus* was detected on 25.3% (*n* = 246/972) of foot-level observations and was detected on all farms and across all visits. Throughout the study duration, 52.1% (*n* = 177/340) of feet had *D. nodosus* detected, where the majority (67.8%, *n* = 120/177) had *D. nodosus* detected at one visit only. Only one foot had *D. nodosus* detected at all four visits. Twenty ewes (23.5%) had no feet with *D. nodosus* detected over the course of the study. No difference in frequency of *D. nodosus* detection between front and back feet was observed (*p* > 0.05). Frequency of *D. nodosus* detection on feet affected by ID was greater than expected by chance (*p* < 0.001) (Table 6).

#### 3.1.5. Load of *Dichelobacter nodosus*

Of swab samples tested positive for *D. nodosus* (detected by 16S rRNA assay), 94.7% (*n* = 233/246) were quantifiable by the *rpoD* qPCR assay. Minimum load detected on feet positive for *D. nodosus* was 0.29 log_10_ + 1 *rpoD* genome copies µL^−1^ and maximum was 5.98 log_10_ + 1 *rpoD* genome copies µL^−1^. Mean load of *D. nodosus* (all feet) was highest on feet with ID compared with healthy feet (*p* < 0.001) (Table 7). Mean load of *D. nodosus* (positive feet only) was highest on feet with ID compared with healthy feet (*p* < 0.001).

### 3.2. Associations with Clinical Status of Feet

Univariable associations with clinical status are found in Supplementary Table S2. AIC and delta AIC values during model selection are found in Supplementary Table S3.

One variable remained in the final model (Table 8). A foot with a higher load of *D. nodosus* had an increased risk of being affected by ID (OR = 4.22, 95% CI: 3.20–5.57). Feet with poor hoof conformation were no more likely to be affected by ID. The model was re-run with variable ‘load of *D. nodosus* for positive feet only’ (omitting feet with undetectable loads) and produced a higher odds ratio (OR = 15.02, 95% CI: 6.12–36.88) with the same significance.

### 3.3. Associations with Presence of Dichelobacter nodosus on Feet

Univariable associations with presence of *D. nodosus* are found in Supplementary Table S4. AIC and delta AIC values during model selection are found in Supplementary Table S5.

Two variables remained in the final model (Table 9). A foot with ID had an increased risk of being positive for *D. nodosus* (OR = 19.13, 95% CI: 9.70–37.73). A foot with wall overgrowth had a reduced risk of being positive for *D. nodosus* (OR = 0.47, 95% CI: 0.26–0.85). Feet with poor sole and heel conformation or wall overgrowth were no more likely to have *D. nodosus* present.

### 3.4. Associations with Load of Dichelobacter nodosus on All Feet

Univariable associations with the load of *D. nodosus* on all feet are found in Supplementary Table S6. AIC and delta AIC values during model selection are found in Supplementary Table S7.

Three variables remained in the final multivariable model for *D. nodosus* load on all feet (Table 10). A foot with ID was more likely to have higher loads of *D. nodosus* (β = 2.53, 95% CI: 2.34–2.72). A foot with poor sole and heel conformation was also more likely to have higher loads of *D. nodosus* (β = 0.19, 95% CI: 0.04–0.35). However, a foot with wall overgrowth was less likely to have higher loads of *D. nodosus* (β = −0.18, 95% CI: −0.34–−0.01).

Univariable associations with the load of *D. nodosus* on positive feet only are found in Supplementary Table S8. AIC and delta AIC values during model selection are found in Supplementary Table S9.

Three variables also remained in the final multivariable model for *D. nodosus* load on positive feet only (Table 11). A positive foot with ID was more likely to have higher loads of *D. nodosus* (β = 1.96, 95% CI: 1.70–2.22). A positive foot with sole and heel damage was also more likely to have higher loads of *D. nodosus* (β = 0.41, 95% CI: 0.15–0.66). Furthermore, a positive foot with wall overgrowth was more likely to have higher loads of *D. nodosus* (β = 0.27, 95% CI: 0.01–0.52).

## 4. Discussion

Investigating the role hoof conformation plays in the infection dynamics of *D. nodosus* is vital to understanding the biological mechanisms affecting host susceptibility and mitigating the welfare and economic costs associated with footrot. This is the first longitudinal study to investigate the presence and load of *D. nodosus* on ovine feet with different hoof conformation traits. We highlight that feet with aspects of poor conformation are likely reservoirs of *D. nodosus* infection and pose considerable risk to the transmission of infection within flocks.

The association between hoof wall overgrowth and *D. nodosus* was found to be multifaceted and complex. Whilst we document the presence of wall overgrowth to be negatively associated with both *D. nodosus* presence and load, on feet positive for *D. nodosus*, wall overgrowth contributes towards an increased load—a novel finding. This indicates that wall overgrowth could facilitate the proliferation of *D. nodosus* once present on the foot. Wall overgrowth covering the sole could create a niche anaerobic environment, so when *D. nodosus* is present, conditions could favour survival and proliferation. These physical niches may also offer protection to *D. nodosus* from circulating antibodies, as previously postulated [32]. Whilst hoof wall overgrowth can be the result of non-weight bearing from previous footrot infection, or due to the diseased hoof horn growing more rapidly [13], we consider *D. nodosus* to have the potential to recrudesce in overgrown feet—a source of re-infection. Our findings provide some further understanding as to why sheep with hoof overgrowth are at increased risk of clinical footrot [33].

Our results suggest that whilst overgrown feet appear clinically healthy, they can act as subclinical carriers of *D. nodosus* and present an important risk for transmission of infection between the feet of sheep when trimming. Overgrown feet could be more prone to accidental over-trimming, resulting in exposure of sensitive tissue and bleeding, which are implicated as risk factors for infectious lameness [2]. Consequently, higher residual loads of *D. nodosus* present on positive feet could increase risk of bacterial entry into underlying tissue. Higher lameness prevalence has been reported in flocks where farmers trimmed misshapen feet without signs of active infection [34]. Our current study provides new evidence to support current industry and veterinary advice to not trim hoof overgrowth, since risks from damage [2] and transmission [35] far outweigh any benefit of removing overgrowth.

Another novel finding was that feet with existing misshapen or damaged sole and heel areas were more likely to have higher loads of *D. nodosus*. This highlights that apparently healthy feet with no active clinical disease or lameness are important reservoirs of *D. nodosus*. Although we cannot imply causality, as the clinical status of feet outside of sampling visits was unknown and episodes of disease may have been missed, it is possible that sole damage, such as necrotising tissue, could arise from a previous episode of SFR. Therefore, elevated loads of *D. nodosus* on feet with poor sole and heel conformation could cause residual infection following the healing of active lesions. This suggests that feet showing apparent recovery from footrot, with no clinical signs of active disease nor lameness, could continue to sequester and spread higher loads of *D. nodosus* into the local environment. This adds weight to previous reports suggesting that sheep with healing footrot lesions may not display locomotion changes [36], and these feet could remain diseased for many months [14]. Whilst recommendations highlight the importance of isolating lame sheep until sound, transmission of *D. nodosus* and repeated episodes of disease could be further mitigated by inspecting feet and ensuring ewes with extensive sole and heel damage remain isolated until tissue heals.

The identification of poor hoof conformation traits associated with increased *D. nodosus* infectiveness has important implications for the persistence of subclinical carriers within flocks. Flock-level *D. nodosus* eradication programmes could be hindered by the presence of subclinical carriers. The economic losses associated with relapse of subclinical carriers, flock re-infection and disease recurrence are difficult to quantify. However, they have considerable emotional costs; sheep farmers feel disheartened by their perceived inability to eliminate lameness [37] and express feelings of hopelessness towards the control of footrot [38].

Whilst the routine surveillance of covert carriers using expensive molecular diagnostics is unlikely in commercial flocks, we highlight the importance of assessing hoof conformation in order to identify sheep at risk of silently spreading *D. nodosus* within the flock. Culling sheep with deformed or misshapen feet, often in hand with chronic lameness and treatment failure, is currently advised in the lameness Five-Point Plan, the UK industry-endorsed programme of practices to reduce lameness in sheep [39]. Rigorous culling of sheep with abnormal hooves, alongside those with clinical symptoms of footrot, has been effective at eliminating virulent strains, but not all strains, of *D. nodosus* in Australian flocks [40]. Breeding replacements from ewes with good hoof conformation could also contribute to increasing flock resilience towards infection, although the heritability of hoof conformation traits have not been fully elucidated [41,42]. Carefully inspecting the feet of purchased sheep, rather than assessing locomotion alone, could also inform quarantine procedures; sheep with damaged sole and heel areas could shed higher loads of novel strains of *D. nodosus* onto the pasture, presenting an important biosecurity risk.

Hoof conformation was not associated with presence of ID in our study. This is unsurprising considering that damage to the interdigital skin is the primary route of *D. nodosus* entry and cause of ID [3,43]. Hoof damage could facilitate the penetration and proliferation of *D. nodosus* into deeper tissue and progression into SFR, but this is unclear in the absence of feet with SFR in our study. This could in part explain why no association between wall damage and *D. nodosus* presence and load was observed. Alternatively, chronic hoof wall damage could expose oxygen to deeper layers of the hoof, which reduces the environment conductive for *D. nodosus* proliferation and disease development [10]. Further investigation into hoof conformation traits and *D. nodosus* virulence strain could be highly informative in characterising the physical niches of virulent and benign strains. Additionally, in flocks with SFR present, further work could investigate the associations between *F. necrophorum* and hoof conformation, considering its role in disease progression [11].

To explore the dynamic relationship between hoof conformation and *D. nodosus*, explanatory variables were lagged to the previous visit, but due to variation in the length of time between visits, results were not considered informative. Furthermore, models were constructed with hoof conformation traits as outcome variables, but neither clinical status nor *D. nodosus* presence and load explained the occurrence of poor hoof conformation. Further investigation is required to elucidate the mechanisms contributing to poor hoof conformation in ewes, essential to minimising production losses from subclinical carriers.

This was a longitudinal, repeated cross-sectional field survey of four commercial UK sheep flocks with a history of footrot. We cannot guarantee that our findings provide a definitive description of the association between *D. nodosus* and hoof conformation that could be generalised to all UK flocks but instead provide an indication of the possible interactions. Environmental factors, such as soil type and local weather conditions, could vary between farms, but the range of farms recruited does provide a reasonable cross-section of lowland UK sheep farms. All observations were recorded by a single observer (CMB) using robust scoring systems to eliminate between-observer bias [44]. Swab samples were also collected by a single researcher (CMB) using standardised protocol across all farms. Due to unforeseen circumstances, storage and processing of swabs from visits 1 and 2 differed to that of visits 3 and 4, but this was accounted for in models by including ‘visit’ as a random effect. Nonetheless, the quantity and quality of genomic DNA extracted from samples processed under different handling methods were consistent. Processing and analysis of swab samples were also performed by a single trained researcher (CMB), although the laboratory protocols conducted did vary from previous studies. Statistical associations found between clinical status and presence and load of *D. nodosus* were consistent with previous literature [10], further emphasising our robust methodology and analysis.

## 5. Conclusions

We report specific morphological characteristics of feet to increase risk of *D. nodosus* infection, updating our epidemiological understanding of *D. nodosus* at foot level. Whilst future investigation is warranted, our findings highlight the alternative hoof phenotypes conductive for *D. nodosus* infection and transmission within flocks. A sophisticated, targeted approach to culling is essential to eliminate *D. nodosus* at flock level, by removing both clinical and subclinical sheep. Hoof conformation scoring systems could be employed by farmers to guide culling and breeding decisions. This study highlights the opportunity to build flock resilience by removing covert reservoirs of infection and susceptible sheep in order to future-proof flocks.

## Figures and Tables

**Table 1:** Characteristics of the farms and flocks (identified A-D) selected for the study.

Farm	Location ^1^	Enterprises	Flock Size ^2^	System	Farmer-Reported Lameness ^3^		
					Average %	Highest %	Causes
A	Wales	Sheep, beef	500	Lowland	3	5	ID, SFR, CODD, SH, TG
B	South West	Sheep, dairy	250	Lowland	3	5	ID, SFR, SH, FA
C	South West	Sheep, beef	540	Lowland	2	4	ID, SFR, SH
D	West Midlands	Sheep, arable	500	Lowland	1	2	ID, SFR

^1^ UK region; ^2^ Number of breeding ewes; ^3^ Farmer-reported lameness in ewes between September 2019 and September 2020; CODD: contagious ovine digital dermatitis; SH: shelly hoof; TG: toe granuloma; FA: foot abscess.

**Table 2:** Four-point ordinal scoring systems for three hoof conformation variables relating to sole and heel, hoof wall and hoof wall overgrowth.

Variable	Description and Coding
Sole and heel	0 = Undamaged sole and heel area with a perfect shape
	1 = Mildly damaged and/or misshapen sole and heel area of the digit (<25%)
	2 = Moderately damaged and/or misshapen sole and heel area of the digit (≥25% to <75%)
	3 = Severely damaged and/or misshapen sole and heel area of the digit (≥75%)
Hoof wall	0 = Undamaged hoof wall with a perfect shape
	1 = Mildly damaged and/or misshapen hoof wall of the digit (<25%)
	2 = Moderately damaged and/or misshapen hoof wall of the digit (≥25% to <75%)
	3 = Severely damaged and/or misshapen hoof wall of the digit (≥75%)
Hoof wall overgrowth	0 = No hoof wall overgrowth
	1 = Mildly overgrown hoof wall covering of the sole (<25%)
	2 = Moderately overgrown hoof wall covering of the sole (≥25% to <75%)
	3 = Severely overgrown wall covering of the sole (≥75%)

**Table 3:** Description and coding of sheep- and foot-level variables considered in analyses.

Variable	Coding and Description
Sheep-level	
Age ^1^	0 = <4 years
	1 = ≥4 years
Body condition score (BCS)	0 = 3.0
	1 = <3.0
	2 = >3.0
Foot-level	
Clinical status	0 = Healthy (no apparent signs of disease)
	1 = ID
Presence of *D. nodosus*	0 = Undetected
	1 = Detected
Load of *D. nodosus* (all feet)	log_10_ + 1 *rpoD* genome copies µL^−1^
Load of *D. nodosus* (positive feet only) ^2^	log_10_ + 1 *rpoD* genome copies µL^−1^
Sole and heel conformation	0 = Good (max score 0)
	1 = Poor (score ≥ 1)
Hoof wall conformation	0 = Good (max score 0)
	1 = Poor (score ≥ 1)
Wall overgrowth	0 = Good (max score 0)
	1 = Poor (score ≥ 1)

^1^ Age of ewe at start of study; ^2^ Omitting feet with undetectable loads.

**Table 4:** Distribution of ewes (*n* = 85) by farm and sampling frequency.

Farm	Ewes	Sampling Frequency			
	*n*	1	2	3	4
A	22	7	1	4	10
B	22	2	11	0	9
C	21	2	8	1	10
D	20	4	2	3	11
Total	85	15	22	8	40

**Table 5:** Frequency distribution of good and poor states of three hoof conformation variables for 972 foot-level observations.

Status	Sole and Heel		Hoof Wall		Wall Overgrowth	
	*n*	%	*n*	%	*n*	%
Good	767	78.9	241	24.8	268	27.6
Poor	205	21.1	731	75.2	704	72.4

**Table 6:** Frequency distribution of *Dichelobacter nodosus* detection by clinical status for 972 foot-level observations.

Status	Healthy		ID Affected	
	*n*	%	*n*	%
Undetected	698	82.3	28	22.6
Detected	150	17.7	96	77.4

**Table 7:** Mean load of *D. nodosus* (log_10_ + 1 *rpoD* genome copies µL^−1^) by clinical status of feet (all feet and feet with *D. nodosus* detected only) from 972 foot-level observations of 85 ewes.

Status	*n*	%	log_10_ + 1 *rpoD* genome Copies µL^−1^ All Feet (SD)	Feet with Quantifiable *D. nodosus*		log_10_ + 1 *rpoD* Genome Copies µL^−1^ Positive Feet Only (SD)
				*n*	%	
Healthy	848	87.2	0.29 (0.71)	137	16.2	1.77 (0.66)
ID	124	12.8	3.04 (2.02)	96	77.4	3.94 (1.31)

SD: standard deviation.

**Table 8:** Binomial mixed effects model of clinical status from 972 foot-level observations.

Variable	*n*	%	Odds Ratio	Lower 95% CI	Upper 95% CI
(Intercept)			0.01	0.00	0.03
Load of *D. nodosus* (all feet)	972	100.0	**4.22**	3.20	5.57
*Random terms*	Variance	SD			
Ewe number	2.47	1.57			
Farm and Visit	1.08	1.04			

Load of *D. nodosus* expressed as log_10_ + 1 *rpoD* genome copies µL^−1^; CI: confidence interval for odds ratio; bold odds ratios are statistically significant at 0.05 as their CIs do not include 1; SD: standard deviation.

**Table 9:** Binomial mixed effects model of presence of *Dichelobacter nodosus* from 972 foot-level observations.

Variable	*n*	%	Odds Ratio	Lower 95% CI	Upper 95% CI
(Intercept)			0.07	0.01	0.36
Clinical status					
Healthy	847	87.1	Ref		
ID	125	12.9	**19.13**	9.70	37.73
Wall overgrowth					
Good (max score 0)	268	27.6	Ref		
Poor (score ≥ 1)	704	72.4	**0.47**	0.26	0.85
*Random terms*	Variance	SD			
Ewe number	0.90	0.95			
Farm and Visit	7.02	2.65			

CI: confidence interval for odds ratio; bold odds ratios are statistically significant at 0.05 as their CIs do not include 1; Ref: baseline category for comparison; SD: standard deviation.

**Table 10:** Linear mixed effects model of load of *Dichelobacter nodosus* on all feet from 972 foot-level observations.

Variable	*n*	%	β	Lower 95% CI	Upper 95% CI
(Intercept)			0.40	0.19	0.61
Clinical status					
Healthy	847	87.1	Ref		
ID	125	12.9	**2.53**	2.34	2.72
Sole and heel					
Good (max score 0)	767	78.9	Ref		
Poor (score ≥ 1)	205	21.1	**0.19**	0.04	0.35
Wall overgrowth					
Good (max score 0)	268	27.6	Ref		
Poor (score ≥ 1)	704	72.4	**−0.18**	−0.34	−0.01
*Random terms*	Variance	SD			
Ewe number	0.11	0.33			
Farm and Visit	0.09	0.29			

β: coefficient; CI: confidence interval for coefficient; bold coefficients are statistically significant at 0.05 as their CIs do not include 0; Ref: baseline category for comparison; SD: standard deviation.

**Table 11:** Linear mixed effects model of the load of *Dichelobacter nodosus* on positive feet only, from 233 foot-level observations.

Variable	*n*	%	β	Lower 95% CI	Upper 95% CI
(Intercept)			1.70	1.38	2.02
Clinical status					
Healthy	137	58.8	Ref		
ID	96	41.2	**1.96**	1.70	2.22
Sole and heel					
Good (max score 0)	164	70.4	Ref		
Poor (score ≥ 1)	69	29.6	**0.41**	0.15	0.66
Wall overgrowth					
Good (max score 0)	114	48.9	Ref		
Poor (score ≥ 1)	119	51.1	**0.27**	0.01	0.52
*Random terms*	Variance	SD			
Ewe number	0.22	0.47			
Farm and Visit	0.08	0.29			

β: coefficient; CI: confidence interval for coefficient; bold coefficients are statistically significant at 0.05 as their CIs do not include 0; Ref: baseline category for comparison; SD: standard deviation.

## Data Availability

The data presented in this study are available on reasonable request from the corresponding author. The data is not publicly available as not all data from the study has been published yet.

## Supplementary Materials

The following are available online at https://www.mdpi.com/article/10.3390/ani11051312/s1, Table S1: Details of the primers and TaqMan^®^ probes. Table S2: Univariable binomial mixed effects model of clinical status from 972 foot-level observations, Table S3: Akaike’s Information Criteria (AIC) and delta AIC during model selection for associations with clinical status, Table S4: Univariable binomial mixed effects model of presence of *D. nodosus* from 972 foot-level observations, Table S5: Akaike’s Information Criteria (AIC) and delta AIC during model selection for associations with presence of *D. nodosus*, Table S6: Univariable linear mixed effects model of load of *Dichelobacter nodosus* on all feet from 972 foot-level observations, Table S7: Akaike’s Information Criteria (AIC) and delta AIC during model selection for associations with load of *D. nodosus* on all feet, Table S8: Univariable linear mixed effects model of load of *Dichelobacter nodosus* on positive feet only from 233 foot-level observations, Table S9: Akaike’s Information Criteria (AIC) and delta AIC during model selection for associations with load of *D. nodosus* on positive feet only.
