# Supplementary material for: Characterisation of Dichelobacter nodosus on Misshapen and Damaged Ovine Feet: A Longitudinal Study of Four UK Sheep Flocks
Source: Animals (Basel). 2021 May 3;11(5):1312. doi: 10.3390/ani11051312 (PMC8147629; doi:10.3390/ani11051312)
Supplement: Supplementary file 1 [file animals-11-01312-s001.zip › animals-1201784-SI.pdf]

Supplementary Materials

Table S1. Details of the primers and TaqMan® probes.

Description	Sequence (5′ to 3′)
16S forward primer	CGGGGTTATGTAGCTTGCTATG
16S reverse primer	TACGTTGTCCCCCACCATAA
16S TaqMan® probe[1]	TGGCGGACGGGTGAGTAATATATAGGAATC-BHQ
rpoD forward primer	GCTCCCATTTCGCGCATAT
rpoD reverse primer	CTGATGCAGAAGTCGGTAGAACA
rpoD TaqMan® probe[2]	CATTCTTACCGGATCCG-BBQ2

[1] 5′ labelled HEX = Hexachloro-fluorescein; [2] 5′ labelled FAM = Fluorescein amidite; BHQ = Black Hole Quencher®-2; BBQ2 = Blackberry® Quencher

Table S2. Univariable binomial mixed effects model of clinical status from 972 foot-level observations.

Variable	*n*	%	Odds ratio	Lower 95% CI	Upper 95% CI	*p*
Sheep-level						
**Ewe age[1] (*n* = 85)**						0.965
<4 years (baseline)	52	61.2	Ref			
≥4 years	33	38.8	0.98	0.36	2.67	
**Ewe BCS (*n* = 243)**						0.063
3.0 (baseline)	111	45.7	Ref			
<3.0	43	17.7	0.36	0.11	1.14	
>3.0	89	36.6	0.40	0.14	1.14	
***Foot-level* (*n* = 972)**						
**Presence of *D. nodosus***						<0.001
Undetected (baseline)	725	74.6	Ref			
Detected	247	25.4	**28.18**	13.34	59.54	
***D. nodosus* load (all samples)**	972	100.0	**4.22**	3.20	5.57	<0.001
***D. nodosus* load (positives only)**	233	100.0	**15.02**	6.12	36.88	<0.001
**Sole and heel damage**						0.024
Good (baseline)	767	78.9	Ref			
Poor	205	21.1	**1.95**	1.09	3.47	
**Wall damage**						0.278
Good (baseline)	241	24.8	Ref			
Poor	731	75.2	1.40	0.76	2.58	
**Wall overgrowth**						0.812
Good (baseline)	268	27.6	Ref			
Poor	704	72.4	0.93	0.49	1.74	

[1]Age of ewe at start of study; Load of *D. nodosus* expressed as $\log_{10}$ +1 *rpoD* genome copies μl^{-1}; CI: confidence interval for odds ratio; Bold odds ratios are statistically significant at 0.05 as their CIs do not include 1; Ref: baseline category for comparison.

Table S3. Akaike's Information Criteria (AIC) and delta AIC during model selection for associations with clinical status.

Model	Variables	AIC	Delta AIC
Final model	Load of *D. nodosus* (all samples)	375.5	
Candidate model 5	Load of *D. nodosus* (all samples)	375.9	0.4
	Sole and heel shape and/or damage		
Candidate model 4	Ewe BCS	378.1	2.6
	Load of *D. nodosus* (all samples)		
	Sole and heel shape and/or damage		
Candidate model 3	Ewe BCS	379.7	4.2
	Load of *D. nodosus* (all samples)		
	Sole and heel shape and/or damage		
	Wall shape and/or damage		
Candidate model 2	Ewe age	381.7	6.2
	Ewe BCS		
	Load of *D. nodosus* (all samples)		
	Sole and heel shape and/or damage		
	Wall shape and/or damage		
Candidate model 1 (full*)	Ewe age	383.7	8.2
	Ewe BCS		
	Load of *D. nodosus* (all samples)		
	Sole and heel shape and/or damage		
	Wall shape and/or damage		
	Wall overgrowth		

*Variable 'presence of *D. nodosus*' not included in full model as highly correlated to variable 'load of *D. nodosus* (all samples)'

Table S4. Univariable binomial mixed effects model of presence of *D. nodosus* from 972 foot-level observations.

Variable	*n*	%	Odds ratio	Lower 95% CI	Upper 95% CI	*p*
Sheep-level						
**Ewe age[1] (*n* = 85)**						0.301
<4 years (baseline)	52	61.2	Ref			
≥4 years	33	38.8	1.40	0.74	2.64	
**Ewe BCS (*n* = 243)**						0.051
3.0 (baseline)	111	45.7	Ref			
<3.0	43	17.7	0.54	0.24	1.19	
>3.0	89	36.6	0.49	0.24	1.02	
Foot-level **(*n* = 972)**						
**Clinical status**						<0.001
Healthy (baseline)	847	87.1	Ref			
ID	125	12.9	**18.13**	9.27	35.48	
**Sole and heel damage**						0.026
Good (baseline)	767	78.9	Ref			
Poor	205	21.1	**1.77**	1.07	2.92	
**Wall damage**						0.931
Good (baseline)	241	24.8	Ref			
Poor	731	75.2	0.98	0.62	1.55	
**Wall overgrowth**						0.033
Good (baseline)	268	27.6	Ref			
Poor	704	72.4	**0.56**	0.33	0.95	

[1]Age of ewe at start of study; CI: confidence interval for odds ratios; Bold odds ratios are statistically significant at 0.05 as their CIs do not include 1; Ref: baseline category for comparison.

Table S5. Akaike's Information Criteria (AIC) and delta AIC during model selection for associations with presence of *D. nodosus*.

Model	Variables	AIC	Delta AIC
Final model	Clinical status	675.2	
	Wall overgrowth		
Candidate model 4	Clinical status	675.2	0.0
	Sole		
	Wall overgrowth		
Candidate model 3	Ewe BCS	676.5	1.3
	Clinical status		
	Sole		
	Wall overgrowth		
Candidate model 2	Ewe age	677.6	2.4
	Ewe BCS		
	Clinical status		
	Sole		
	Wall overgrowth		
Candidate model 1 (full)	Ewe age	679.2	4.0
	Ewe BCS		
	Clinical status		
	Sole		
	Wall		
	Wall overgrowth		

Table S6. Univariable linear mixed effects model of load of *Dichelobacter nodosus* on all feet from 972 foot-level observations.

Variable	*n*	%	β	Lower 95% CI	Upper 95% CI	*p*
Sheep-level						
**Ewe age[1] (*n* = 85)**						0.546
<4 years (baseline)	52	61.2	Ref			
≥4 years	33	38.8	-0.09	-0.38	0.20	
**Ewe BCS (*n* = 243)**						0.059
3.0 (baseline)	111	45.7	Ref			
<3.0	43	17.7	-0.29	-0.60	0.02	
>3.0	89	36.6	-0.21	-0.46	0.04	
***Foot-level* (*n* = 972)**						
**Clinical status**						<0.001
Healthy (baseline)	847	87.1	Ref			
ID	125	12.9	**2.55**	2.36	2.74	
**Sole and heel damage**						<0.001
Good (baseline)	767	78.9	Ref			
Poor	205	21.1	**0.37**	0.17	0.57	
**Wall damage**						0.396
Good (baseline)	241	24.8	Ref			
Poor	731	75.2	0.08	-0.11	0.27	
**Wall overgrowth**						0.162
Good (baseline)	268	27.6	Ref			
Poor	704	72.4	-0.16	-0.37	0.06	

[1]Age of ewe at start of study; β: coefficient; CI: confidence interval for coefficient; Bold coefficients are statistically significant at 0.05 as their CIs do not include 0; Ref: baseline category for comparison.

Table S7. Akaike's Information Criteria (AIC) and delta AIC during model selection for associations with load of *D. nodosus* on all feet.

Model	Variables	AIC	Delta AIC
Final model	Clinical status	2644.5	
	Sole and heel shape and/or damage		
	Wall overgrowth		
Candidate model 3	Ewe BCS	2650.7	6.2
	Clinical status		
	Sole and heel shape and/or damage		
	Wall overgrowth		
Candidate model 2	Ewe age	2654.8	10.2
	Ewe BCS		
	Clinical status		
	Sole and heel shape and/or damage		
	Wall overgrowth		
Candidate model 1 (full)	Ewe age	2660.1	15.6
	Ewe BCS		
	Clinical status		
	Sole and heel shape and/or damage		
	Wall shape and/or damage		
	Wall overgrowth		

Table S8. Univariable linear mixed effects model of load of *Dichelobacter nodosus* on positive feet only from 233 foot-level observations.

Variable	*n*	%	β	Lower 95% CI	Upper 95% CI	*p*
Sheep-level						
**Ewe age[1] (*n* = 65)**						0.085
<4 years (baseline)	44	67.7	Ref			
≥4 years	21	32.3	-0.58	-1.24	0.08	
**Ewe BCS (*n* = 95)**						0.465
3.0 (baseline)	48	50.5	Ref			
<3.0	14	14.7	-0.28	-0.90	0.33	
>3.0	33	34.7	-0.21	-0.72	0.30	
***Foot-level* (*n* = 233)**						
**Clinical status**						<0.001
Healthy (baseline)	137	58.8	Ref			
ID	96	41.2	**1.98**	1.71	2.25	
**Sole and heel damage**						0.003
Good (baseline)	164	70.4	Ref			
Poor	69	29.6	**0.48**	0.16	0.80	
**Wall damage**						0.1038
Good (baseline)	63	27.0	Ref			
Poor	170	73.0	0.28	-0.06	0.62	
**Wall overgrowth**						0.173
Good (baseline)	114	48.9	Ref			
Poor	119	51.1	0.17	-0.21	0.55	

[1]Age of ewe at start of study; β: coefficient; CI: confidence interval for coefficient; Bold coefficients are statistically significant at 0.05 as their CIs do not include 0; Ref: baseline category for comparison.

Table S9. Akaike's Information Criteria (AIC) and delta AIC during model selection for associations with load of *D. nodosus* on positive feet only.

Model	Variables	AIC	Delta AIC
Final model	Clinical status	614.0	
	Sole and heel shape and/or damage		
	Wall overgrowth		
Candidate model 3	Ewe age	615.2	1.2
	Clinical status		
	Sole and heel shape and/or damage		
	Wall overgrowth		
Candidate model 2	Ewe age	618.6	4.7
	Clinical status		
	Sole and heel shape and/or damage		
	Wall shape and/or damage		
	Wall overgrowth		
Candidate model 1 (full)	Ewe age	624.7	10.7
	Ewe BCS		
	Clinical status		
	Sole and heel shape and/or damage		
	Wall shape and/or damage		
	Wall overgrowth		
